# Efficient Management of Asbestos Waste Through Utilization as Mineral Additives in Portland Cement Production

**DOI:** 10.3390/ma17235793

**Published:** 2024-11-26

**Authors:** Karol Durczak, Michał Pyzalski, Agnieszka Sujak, Michał Juszczyk, Dariusz Sala, Leonas Ustinovichius

**Affiliations:** 1 Department of Biosystems Engineering, Faculty of Environmental and Mechanical Engineering, Poznan University of Life Sciences, Wojska Polskiego 50, 60-627 Poznan, Poland; karol.durczak@up.poznan.pl (K.D.); agnieszka.sujak@up.poznan.pl (A.S.); 2 Faculty of Materials Science and Ceramics, AGH University of Krakow, Al. Mickiewicza 30, 30-059 Krakow, Poland; 3 Faculty of Civil Engineering, Cracow University of Technology, 31-155 Cracow, Poland; michal.juszczyk@pk.edu.pl; 4 Department of Enterprise Management, AGH University of Krakow, Al. Mickiewicza 30, 30-059 Krakow, Poland; sala@agh.edu.pl; 5 Institute of Sustainable Construction, Faculty of Civil Engineering, Vilnius Gediminas Technical University, 10223 Vilnius, Lithuania; leonas.ustinovicius@vilniustech.lt

**Keywords:** supplementary cementitious materials, asbestos, waste utilization, neutralization methods, recycling, health safety, environmental protection, sustainable development, sustainable development, synthesis and preparation

## Abstract

This article presents research on the effectiveness of utilizing asbestos waste, particularly chrysotile asbestos, in the production of Portland cement. The study aimed to evaluate the feasibility of transforming asbestos cement (Eternit) through thermal treatment and its enrichment with mineral additives, enabling its integration into the clinker synthesis process. Differences in the physicochemical properties of types of cement produced from conventional raw materials and those manufactured using asbestos waste were analyzed. The research findings indicate that the presence of asbestos in cementitious materials leads to a significant mass loss of 29.4% due to thermal decomposition. Chemical analysis revealed the presence of aluminum oxide (Al_2_O_3_) and iron oxide (Fe_2_O_3_) at levels of 4.10% and 3.54%, respectively, suggesting the formation of brownmillerite, a phase typical of cement clinker. Furthermore, compressive strength tests on asbestos-modified cements demonstrated comparable mechanical properties to reference cement (CEM I), indicating their potential applicability in construction. This study provides essential insights into the mineralogical composition of asbestos cement, which is crucial for developing effective methods for its safe disposal. It represents a significant step toward sustainable asbestos waste management and the promotion of innovative solutions in the construction industry.

## 1. Introduction

Asbestos refers to a group of silicate minerals that form fibrous structures. Under natural conditions, six primary asbestos minerals are distinguished, which are categorized into two main groups: serpentines and amphiboles. The serpentine group includes chrysotile, which is the most commonly used in industry and constitutes 90% of global asbestos production [1]. Chrysotile is distinguished by its unique crystalline structure, composed of alternating tetrahedral and octahedral layers. The tetrahedral layers consist of silicate networks (SiO_4_) arranged in a pseudo-hexagonal configuration, while the octahedral layers are made up of magnesium ions (Mg^2^+) and hydroxyl groups (OH^−^). The combination of these two types of layers in a 1:1 ratio forms the fundamental structural unit of chrysotile. Due to the differences in layer dimensions (with the octahedral layers being slightly larger than the tetrahedral ones), the structure experiences strain, which is relieved by the rolling of the layers around the fiber axis. This results in a tubular structure resembling a rolled papyrus or a spiral. Such a configuration provides chrysotile with flexibility and the ability to withstand stress without permanent structural damage [2]. Due to these properties, chrysotile fibers can be processed, rendering it a highly versatile material.

The amphibole group includes minerals such as actinolite, amosite, anthophyllite, crocidolite, and tremolite. Amphiboles are characterized by a fibrous, chain-like structure. They are harder and more brittle than chrysotile, which affects their physical properties and industrial applications. Asbestos is valued for its exceptional physical properties, including high tensile strength, low thermal conductivity, resistance to chemical degradation, and sound absorption capability. These attributes make asbestos highly valuable across various industrial sectors, particularly in construction.

In former times, asbestos was widely used in insulation materials, plasters, paints, roofing, pipes, and many other construction products as a cost-effective and easy-to-process material [3]. Its content could range from 20% to over 95% by mass. Unfortunately, exposure to asbestos fibers leads to severe diseases such as lung cancer, mesothelioma, and asbestosis [4]. Annually, the death toll from these conditions exceeds one hundred thousand [5]. Asbestos fibers are hazardous because they can enter the respiratory system, where they may trigger serious diseases. Fibers measuring 5–10 μm in length and between 0 and 1 μm in diameter are extremely dangerous. Health issues related to asbestos have become a global challenge, affecting not only industrial workers but also residents living near industrial sites [6].

The International Labour Organization (ILO) focuses on health protection related to asbestos, implementing a global ban on the use of asbestos as well as products and waste containing this material. In many countries, asbestos has been completely banned, and asbestos waste must be disposed of and managed by stringent safety regulations [7]. The removal of asbestos-containing materials generates toxic waste and entails high costs. While landfill disposal is commonly practiced, it does not represent a definitive solution to the problem.

In 2013, during extensive discussions in the European Parliament, a decision was made to adopt asbestos inertization technology as an effective solution to the problem. The inertization process involves transforming asbestos into a harmless material and stabilization, which aims to reduce fiber emissions without altering the material’s crystalline structure or by entirely converting asbestos fibers into non-hazardous compounds. Inertization technologies can be divided into three main categories: thermal, chemical, and mechanochemical methods [8]. Thermal methods utilize heat to transform asbestos into an inert material at temperatures where fibers lose stability. Chemical methods involve reactions that convert asbestos into non-hazardous compounds. Meanwhile, mechanochemical methods rely on the mechanical grinding of asbestos fibers. Each of these methods has its advantages and drawbacks, yet all aim to eliminate the risks associated with asbestos, providing a safe and effective solution to asbestos waste [9]. Among asbestos waste disposal methods, thermal treatment stands out as particularly promising [10].

This process, involving the transformation of asbestos under high-temperature conditions, offers several advantages, including minimizing the release of harmful fibers into the environment. During thermal treatment, asbestos undergoes dehydroxylation, which leads to its conversion into more stable mineral forms or complete melting through vitrification. This approach not only effectively eliminates the hazardous properties of asbestos but can also contribute to reducing the volume of waste in the environmental cycle.

In the context of asbestos waste utilization, one promising direction is its application as a mineral additive in the production of Portland cement [11]. The integration of processed asbestos waste with Portland cement could offer benefits in terms of both production efficiency and environmental protection. The use of asbestos waste in cement products not only enables the effective removal of hazardous fibers from circulation but may also help reduce the demand for natural raw materials and lower emissions associated with cement production [12].

Although the thermal treatment method presents certain challenges, such as operational costs and the energy intensity of the process, its potential ecological and economic benefits are substantial. In particular, integrating thermal technologies with renewable energy sources and further may help reduce costs and increase process efficiency. This approach represents an important step toward sustainable development, offering a comprehensive solution to asbestos waste disposal and making a significant contribution to environmental protection [13].

The focus of this study was to evaluate the effectiveness of utilizing processed asbestos (chrysotile) waste in Portland cement production. The research centers on the application of thermal treatment to transform asbestos cement (Eternit) waste, which is then enriched with mineral additives and incorporated into thermal synthesis processes for inclusion in cement clinker composition. The goal was to examine the physicochemical property differences between types of cement produced from conventional raw materials and those derived from chrysotile waste.

## 2. Methods

### 2.1. Cement Synthesis

**Figure 1:**
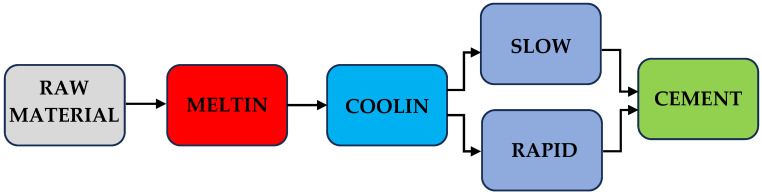
Diagram of cement synthesis from modified asbestos cement waste.

The cement was obtained by melting asbestos cement waste with the addition of corrective raw materials. The cement production in the planned experiment followed two variants: the first involved slow cooling, and the second involved rapid cooling of the molten samples, as illustrated in Figure 1.

The melting process was conducted on cylinders with a diameter of 50 mm and a height of 80 mm, placed in platinum crucibles. The synthesis was carried out in a Superkanthal furnace. The synthesis cylinders were prepared from a homogeneous raw mix, initially moistened with water (7–8%), then molded into cylinders, compacted using a hydraulic press, and dried at 110 °C. The melting process involved heating the samples to a temperature 20 °C higher than the characteristic temperature cited in the literature [14]. The molten materials were held at maximum temperature for 1 h. Two series of samples were prepared: one cooled slowly and the other rapidly. Rapid cooling involved immediately removing the crucibles with samples from the furnace and placing them in a water bath at 10 °C. Slow cooling involved leaving the molten material in the furnace, cooling it at a rate of about 5 °C/min down to 1000 °C, and then at about 20 °C/min down to 400 °C. Once this temperature was reached, the samples were removed from the furnace and left to cool to room temperature. In the next stage, the samples were pre-crushed in an agate mortar and then ground in a vibratory mill until particles reached a size corresponding to a 0.063 mm sieve mesh. The same grinding conditions were applied to all samples. The oxide content in the obtained samples was determined in accordance with the PN-EN 196-2:2006 standard [15].

### 2.2. XRD Analysis

Qualitative and quantitative phase composition analysis was conducted using X-ray diffraction (XRD) on samples with particle sizes below 0.063 mm. A vibratory agate mill (Frisch, Idar-Oberstein, Germany) was used to grind 2 g of the samples. For quantitative analysis, 15% by weight of metallic silicon was added to the samples as a standard. The grinding time was 2 h, with a mill amplitude of 0.2 mm and a vibration frequency of 2000 Hz. Both standard-free and standard-added samples were prepared identically. The ground samples, approximately 1 g each, were placed in a flat measurement holder and immediately measured to minimize moisture exposure.

XRD measurements were carried out using a “PHILIPS” apparatus (Amsterdam, The Netherlands), consisting of an X-ray tube power supply PW1140/00/60 and an upgraded PW 1050/50 vertical goniometer (Philips, Amsterdam, The Netherlands). The goniometer was equipped with software for fully automatic control and simultaneous digital recording of measurement results. The device used a vertically mounted “PHILIPS” X-ray tube with a copper anticathode (Cu). Measurements were performed within a fixed angular range from 10° to 80°.

For quantitative studies, a 15%-by-weight addition of powdered metallic silicon, prepared under the same conditions as the other samples, was used. Based on the X-ray data, qualitative analysis was conducted to identify the phases present, as well as quantitative analysis to determine the mass proportions of crystalline and amorphous phases and the unit cell parameters of the identified phases. Quantitative analysis employed the Rietveld method [16], with data processed using specialized software such as X’Pert HighScore Plus v. 2.1.

### 2.3. High-Temperature Microscopy

To determine the characteristic temperature of thermal treatment for the asbestos cement sample, an analysis was conducted using an EM301 heating microscope (Hesse Instruments e. K., Osterode am Harz, Germany). The sample was examined optically using a non-contact technique at temperatures reaching a maximum of 1400 °C, with a heating rate of 5 °C/min. The EMI III software is 3.1.3 integrated with the device automatically analyzed the sample’s silhouettes, determining its geometric parameters and characteristic temperatures during heating. The design of the measurement chamber allowed the sample to be heated beyond its melting point, enabling precise determination of the material’s properties [17].

The analysis investigated the sample’s characteristics such as sintering behavior, softening characteristics, melting behavior, thermal expansion, and wettability. The results obtained provided detailed information on the thermal properties of the asbestos cement sample.

### 2.4. Compressive Strength of Cements

Studies on the mechanical strength variations of cements produced from asbestos cement waste and reference cement (CEM I) were conducted. Compressive strength tests were performed using a hydraulic compact press (Controls S.p.A., Milan, Italy). The tests were carried out in accordance with the PN-EN 196-1 standard [18]. Samples were prepared and tested under standard laboratory conditions. The results enabled a comparison of the mechanical properties of asbestos-modified cement with the reference cement, allowing for an assessment of its potential applications.

### 2.5. Studies on the Shrinkage and Expansion of Cements

The potential shrinkage and expansion of the cements were evaluated using the American standard ASTM C 845–96 [19], which provides guidelines for classifying expansive and non-shrinkage cements as well as methodologies for testing material properties and requirements. Additional observations and calculations were carried out using methods available in the authors’ laboratory. The initial water demand and setting time of the binders were determined using a Vicat apparatus. Dimensional changes were measured on standard 40 × 40 × 160 mm mortar beams with empirically defined water-to-cement ratios, following PN-EN 196-1. Free expansion tests, in accordance with ASTM C 845–96, were conducted using a Graf-Kaufmann apparatus [20].

Analyses of phase composition and mechanical strength complemented standard performance tests. Statistical analysis involved calculating the arithmetic mean of three measurements taken at specified intervals as outlined in the research plan. Linear changes in the samples were assessed 15 times, with the first day of hydration serving as the baseline. Fourteen measurements were conducted over the first 28 days (on days 1–3, 6–7, 10, 14–17, 20–21, 24, and 28), and a final measurement was performed after 90 days to evaluate the binding process of the cement mortar.

## 3. Results and Discussion

### 3.1. Results of Chemical Analysis of Raw Materials

For the experimental studies, asbestos cement waste (Eternit) was randomly sampled from storage facilities located in southern Poland. Calcium carbonate and silica gel, characterized by high purity and suitable for analytical applications, were used as corrective raw materials. A detailed chemical analysis of the raw materials used is presented in the tables below. The chemical analysis was performed in accordance with the PN-EN 196-2 standard [15,20]. Additionally, Portland cements CEM I 42.5 R and N were included in the study as reference samples compared to the cement derived from asbestos waste.

Table 1 presents the chemical analysis of a calcium carbonate (CaCO_3_) sample, revealing that this material consists predominantly of pure calcium carbonate, comprising up to 99% by mass. This high level of purity suggests that the material is suitable for applications requiring minimal impurity content. Acid-insoluble substances in hydrochloric acid (HCl) are present only at 0.005%, indicating that most of the sample dissolves in an acidic environment, which is typical for pure calcium carbonate. The total nitrogen (N) content is 0.04%, a relatively low value, possibly indicating trace amounts of organic or nitrogenous compounds in the material. Chlorides (Cl) are negligible, at 0.001%, indicating a low presence of chloride salts in the sample. Sulfates (SO_4_) are present at 0.05%, also reflecting trace sulfate levels consistent with low-impurity content. The analysis also revealed minor amounts of magnesium (Mg) and sodium (Na) at 0.05% each, suggesting trace amounts of these elements, possibly from natural mineral impurities. Potassium (K) is present in trace amounts at 0.01%, and iron (Fe) at 0.001%, indicating minimal concentrations of these elements, which do not affect the quality of calcium carbonate. The calcium carbonate sample exhibits very high purity, with minimal impurities, making it suitable for applications requiring high-quality mineral raw materials.

Table 2 presents the chemical analysis of silica gel, which reveals that the primary component of the material is silicon dioxide (SiO_2_), constituting 99.2% by mass, indicating the high purity of the gel. Metallic impurities, such as sodium oxide (Na_2_O) at 0.12%, aluminum oxide (Al_2_O_3_) at 0.07%, and iron oxide (Fe_2_O_3_) at 0.05%, fall within acceptable limits for this type of silica gel, confirming that the product is of good quality and suitable for analytical purposes. The presence of titanium dioxide (TiO_2_) is marginal, below 0.01%, further supporting its purity. Moisture content is 0.15%, indicating a small amount of water physically adsorbed on the surface and within the gel’s pores. Loss on ignition, measured at 1.44% at 1000 °C, includes both the loss of adsorbed and chemically bound water, as well as potentially minimal amounts of organic impurities. This level of loss on ignition is typical for silica gel and indicates the presence of some water as an integral part of the gel structure.

The chemical analysis of the asbestos cement sample, presented in Table 3, reveals its complex mineral composition. The dominant chemical component in the sample is calcium oxide (CaO), with a content of 56.86%. This is the primary component of various calcium minerals, indicating the presence of calcium carbonate (CaCO_3_), a key mineral phase in this sample. Calcium carbonate is a fundamental component of raw materials such as limestone and may also derive from cementitious materials commonly used in asbestos cement production. The high CaO content also suggests the presence of calcium hydroxide (Ca(OH)_2_), which may form due to the hydration of calcium oxide, particularly as the asbestos cement ages, influencing its alkalinity. The presence of silica (SiO_2_) at 25.83% indicates a significant amount of silicon within the sample structure, primarily in the form of quartz (SiO_2_). Quartz imparts hardness and mechanical strength to the material, and its presence is typical of many construction materials. Additionally, the combination of calcium and silicon oxides suggests the possible presence of alite (Ca_3_SiO_5_, also known as C_3_S), a characteristic mineral phase of Portland cement. Alite is responsible for material strength in the initial hydration stages and may indicate the use of cement in the production of asbestos cement. Magnesium oxides (MgO), with a content of 6.93%, also play a significant role in the sample composition and are strongly associated with the presence of chrysotile (Mg_3_Si_2_O_5_(OH)_4_), a primary component of asbestos. Chrysotile is a fibrous form of magnesium silicate commonly used in asbestos cement production due to its durability and resistance to external factors [14,20].

Unfortunately, its presence is also associated with health risks due to the release of asbestos fibers into the environment, making asbestos cement a hazardous material. Additionally, the chemical analysis identified aluminum oxide (Al_2_O_3_) and iron oxide (Fe_2_O_3_) at levels of 4.10% and 3.54%, respectively, suggesting the presence of the brownmillerite phase (Ca_2_AlFeO_5_). Brownmillerite is a typical phase in cement clinker, responsible for some hydration properties of the material, although it does not significantly impact its mechanical strength. Another important chemical component is sulfur trioxide (SO_3_), with a content of 1.7%. The high sulfur content suggests the presence of gypsum (CaSO_4_·2H_2_O), which may be related to hydration processes or added as a setting regulator. Gypsum affects the physical properties of asbestos cement, particularly in terms of moisture resistance and structural stability. The chemical analysis of asbestos cement reveals the presence of mineral phases such as calcium carbonate (CaCO_3_), calcium hydroxide (Ca(OH)_2_), alite (Ca_3_SiO_5_), chrysotile (Mg_3_Si_2_O_5_(OH)_4_), quartz (SiO_2_), brownmillerite (Ca_2_AlFeO_5_), and gypsum (CaSO_4_·2H_2_O). These phases contribute to both the material’s strength properties and its susceptibility to degradation, as well as to health hazards associated with the presence of asbestos (Table 3).

The initial preparation of raw materials involved gathering 3 kg of each of the three components, which were then homogenized for 36 h to ensure uniform composition. After the homogenization process, each portion was dried at 110 °C for 24 h to remove excess moisture. After drying, the raw materials were securely sealed in 10 L plastic containers to preserve them for subsequent processing stages.

Representative samples were taken from the prepared portions to determine their loss on ignition. This process was conducted at 1000 °C for 2 h. Loss on ignition, representing mass loss due to the evaporation of water and volatile compounds, allows for the assessment of how each raw material changes in mass as a result of thermal treatment. The results of these analyses are as follows:

- CaCO_3_—43.73%, due to the decomposition of calcium carbonate into calcium oxide (CaO) and carbon dioxide (CO_2_),
- SiO_2_—1.44%, indicating minimal losses primarily associated with the presence of adsorbed moisture or minor impurities,
- Asbestos cement—29.4%, suggesting significant mass loss due to the thermal decomposition of asbestos and other volatile components in the material.

Based on the obtained loss on ignition results, appropriate calculations were conducted to precisely weigh the individual components, accounting for the altered mass of raw materials after calcination. The prepared portions of materials were placed in airtight 2 L plastic containers, and each was supplemented with 7 mm stainless steel balls to enhance the mixing process and prevent secondary segregation of the components. The entire homogenization process lasted 72 h. These prepared raw materials served as the starting material for further studies on the thermal synthesis of modified cement clinkers. Considering the loss on ignition was crucial for accurately determining the proportions of components, directly impacting the efficiency of the synthesis process and the quality of the final product.

The chemical compositions of three different CEM I 42.5 Portland cements produced by the “Chełm”, “Górażdże”, and “Odra” plants are presented in Table 4. The chemical analysis results show differences in the main mineral component content. These cements primarily contain calcium oxide (CaO), the largest component in their composition, ranging from 64.00% to 64.90%. This is a key component responsible for the cement’s primary hydraulic properties. The cement produced by “Górażdże” has the highest CaO content, while “Odra” cement contains slightly less. The second important component is silica (SiO_2_), with content ranging from 18.50% to 21.48%. “Odra” cement has the lowest silica content (18.50%), which may affect its physical properties, particularly the development of strength over time.

“Górażdże” cement contains the highest level of SiO_2_ (21.48%), which suggests it may exhibit improved mechanical properties related to long-term strength. Aluminum oxide (Al_2_O_3_) in these cements ranges from 3.23% to 5.50%. “Chełm” cement contains the least Al_2_O_3_ (3.23%), while “Odra” cement has the highest content (5.50%). A high level of Al_2_O_3_ in cement can contribute to faster setting, which is advantageous in construction work where a quick reaction of the cement is required. Another key component is iron oxide (Fe_2_O_3_), present in amounts from 2.70% to 4.51%. “Chełm” cement has the highest Fe_2_O_3_ content, while “Odra” cement contains the least. Fe_2_O_3_ is responsible for the clinker color and, to some extent, enhances cement resistance to high temperatures [20]. The magnesium oxide (MgO) content in these cements ranges from 1.13% to 1.60%.

The lowest MgO content is found in “Chełm” cement (1.13%), while “Odra” has the highest (1.60%). MgO can affect concrete durability, as excessive amounts may lead to expansion and cracking during concrete hardening [20]. Sulfur trioxide (SO_3_) is present in amounts ranging from 2.10% to 3.08%. The highest SO_3_ content was recorded in “Chełm” cement (3.08%), which can influence bonding capacity and chemical resistance in concrete, while “Odra” cement contains the least (2.10%). Potassium oxide (K_2_O) content varies from 0.43% to 1.00%, with “Chełm” cement having the lowest (0.43%) and “Odra” the highest (1.00%). Alkaline oxides such as K_2_O can impact the alkali–silica reaction in concrete, which is important for its long-term durability [20]. Sodium oxide (Na_2_O) is present in relatively small amounts, from 0.10% to 0.20%. The highest Na_2_O level was found in “Chełm” cement (0.20%), potentially affecting the cement’s alkaline properties. “Odra” cement has the lowest Na_2_O content (0.10%). Chlorides (Cl^−^) are present in amounts from 0.028% to 0.08%. The highest chloride content is noted in “Górażdże” cement (0.08%), while “Odra” cement has the least (0.028%). The presence of chlorides in cement is significant in the context of reinforcement corrosion in concrete, as higher chloride levels may adversely affect structural durability [20].

The sodium oxide equivalent content (Na_2_Oeq), an important indicator of the impact of alkaline oxides on reactions in concrete, is provided only for “Chełm” cement, where it is 0.48%. This value is not reported for “Górażdże” and “Odra” cements.

The cements analyzed have similar chemical compositions, but variations in the proportions of specific oxides may influence the particular properties of concrete made from these cements, such as setting time, chemical resistance, durability, and long-term strength. Therefore, “Chełm”, “Górażdże”, and “Odra” cements can be selected based on the technical requirements of a specific construction project.

### 3.2. Results of High-Temperature Microscopy

**Figure 2:**
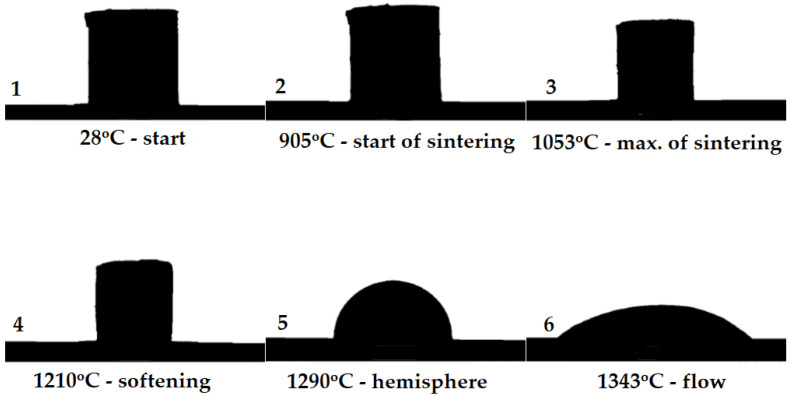
Stages of asbestos cement sample deformation during heating, illustrated using high-temperature microscopy. (**1**—start of heating; **2**—start of sintering; **3**—max. of sintering; **4**—softening; **5**—hemisphere; **6**—flow).

In the experiment, changes in the shape of the sample were observed during heating in the high-temperature microscope. These changes are illustrated in Figure 2. At the initial temperature of 28 °C, the sample had a cylindrical shape with straight, distinct edges, indicating an intact state before the onset of thermal processes.

As the temperature increased to 905 °C, the onset of sintering was observed, evidenced by a slight rounding of the edges at the base of the sample, indicating the beginning of structural changes in the material. Upon reaching 1053 °C, the material reached the peak sintering stage. The sample retained a shape close to a cylinder, though its edges were more rounded, suggesting further densification and consolidation of grains (Figure 2).

At 1210 °C, the material exhibited noticeable softening, resulting in a significant reduction in sample height and a more rounded top. This indicates that the material entered a plastic phase, losing its original rigidity.

At 1290 °C, the sample assumed a hemispherical shape, characteristic of a material reaching a soft state, though not yet fully fluid. In this phase, the material reaches an equilibrium between surface tension and gravity, adopting a rounded shape.

Finally, at 1343 °C, the material entered the flow phase, leading to further deformation of the sample, which became almost flat. This is due to the transition of the material to a fluid or semi-fluid state, where it can no longer maintain its structure, resulting in a complete loss of its original form.

Thus, the processes observed in the experiment indicate typical stages of thermal transformation in the material: from a solid state through softening to flow, which aligns with the expected physical changes occurring during the heating of solids to high temperatures. The presented graph, which correlates with the previously described image, shows the percentage changes in the sample surface area as a function of temperature, reflecting the shape changes as the temperature increases in high-temperature microscopy.

The horizontal axis represents temperature (in °C), while the vertical axis represents the percentage change in the sample surface area. At the beginning of the graph, from the initial temperature up to around 900 °C, the sample surface remains stable at approximately 100%, corresponding to the period during which the sample shape does not undergo visible deformation.

Around 900 °C, a sharp decrease in the sample surface area is observed, corresponding to the onset of the sintering process (the stage observed in the image at 905 °C). This decline continues up to a temperature around 1050 °C, marking maximum sintering, where the sample surface area significantly reduces, indicating material densification. Then, from approximately 1050 °C to 1200 °C, the decrease in surface area becomes more gradual, associated with further structural changes, such as the beginning of sample softening.

At around 1210 °C, according to the graph, the sample surface begins to decrease more slowly; however, near 1290 °C, a sudden increase in surface area occurs, which can be correlated with the sample adopting a hemispherical shape, as observed in the image. This increase in surface area is related to the deformation of the sample into a hemispherical shape, causing a temporary rise in surface area on the graph.

Finally, upon reaching approximately 1340 °C, a further sharp decrease in surface area occurs, corresponding to the sample’s flow stage, where the material becomes fluid and can no longer maintain its shape. This reduction in surface area results from the flattening of the material as it transitions to a semi-fluid or fluid state. This graph illustrates the changes in the sample’s surface area at various thermal stages, from a stable solid state, through sintering, softening, and finally to flow, directly correlating with the processes observed in the previously presented image [21].

### 3.3. Chemical Analysis of Clinker

The modified raw material samples intended for cement clinker production using asbestos cement waste are presented in Table 5. Three different formulations with varying proportions of components were used in the experiment. In each case, asbestos cement constitutes the largest percentage of the composition, ranging from 65% in the first sample to 85% in the third. Calcium oxide (CaO) is the second most abundant component, decreasing proportionally from 30% to 15%, while silicon dioxide (SiO_2_) is present in smaller amounts, only in two samples, decreasing from 5% to 3%.

In each case, asbestos cement constitutes the largest percentage of the composition, ranging from 65% in the first sample to 85% in the third. Calcium oxide (CaO) is the second most abundant component, decreasing proportionally from 30% to 15%, while silicon dioxide (SiO_2_) is present in smaller amounts, at 5% and 3%, respectively.

**Figure 3:**
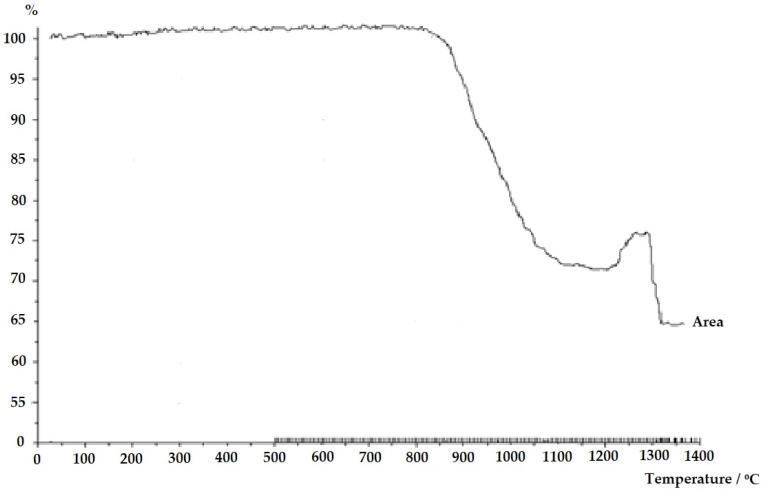
Graph of sample surface area changes as a function of temperature during the heating process.

In the study, two series of samples were prepared and subjected to different cooling processes: slow and rapid (Figure 3). In the next stage, the samples were pre-crushed in an agate mortar (Conbest, Kraków, Poland) and then ground in a vibratory mill to obtain particle sizes corresponding to a 0.01 mm sieve mesh (Frisch GmbH & Co KG, Munich, Germany). Identical grinding parameters were applied to all samples. The chemical composition of the obtained samples was analyzed in accordance with the PN-EN 196-2:2006 standard [15], and the detailed results of this analysis are presented in Table 6.

The chemical composition analysis of samples subjected to different cooling methods reveals significant differences in the content of individual oxides, resulting from the influence of these processes on the material’s microstructure.

In terms of SiO_2_ content, rapidly cooled samples exhibited relatively stable values, ranging from 19.99% to 21.29%. In contrast, slowly cooled samples showed greater variation in SiO_2_ content, with values ranging from 18.84% to 21.44%. This suggests that a slower cooling process promotes a more heterogeneous redistribution of silica, whereas rapid cooling allows for a more uniform distribution of silica within the structure.

The Al_2_O_3_ content shows a clear tendency toward higher values in rapidly cooled samples, reaching levels from 4.18% to 6.27%, while in slowly cooled samples, its content decreases, with values between 2.22% and 5.39%. This indicates greater retention of aluminum oxide in the structure of rapidly cooled samples, possibly due to reduced segregation potential during rapid cooling of the melt.

Similar trends can be observed for Fe_2_O_3_. Rapidly cooled samples contain higher concentrations of this oxide, ranging from 1.68% to 3.14%, compared to slowly cooled samples, in which Fe_2_O_3_ content is lower, ranging from 1.43% to 2.42%. This may suggest that faster cooling limits the segregation process of iron oxides, resulting in a higher Fe_2_O_3_ content in the final product.

The TiO_2_ content shows only minimal differences depending on the cooling method, with rapidly cooled samples containing slightly higher concentrations of this oxide (0.18% to 0.24%) compared to slowly cooled samples (0.12% to 0.23%). This indicates that the cooling process has little impact on this component, likely due to its chemical stability and lower sensitivity to changes during crystallization.

The analysis of CaO content reveals that slow cooling promotes a higher concentration of this oxide in the samples. CaO values in slowly cooled samples range from 68.62% to 69.38%, whereas in rapidly cooled samples, CaO content is lower, ranging from 62.20% to 67.19%. Higher values in slowly cooled samples may result from more uniform CaO crystallization during the extended cooling process.

Finally, the MgO content shows noticeable differences depending on the cooling method. Rapidly cooled samples exhibit higher MgO concentrations, ranging from 3.75% to 9.67%, while slowly cooled samples contain lower amounts of this oxide, between 2.68% and 5.65%. This suggests that rapid cooling promotes the retention of higher MgO concentrations within the sample structure, possibly due to quicker cessation of chemical reactions during cooling.

The differences in oxide content depending on the cooling method indicate a clear influence of cooling process parameters on the chemical composition of the samples. Faster cooling favors higher retention of aluminum, iron, and magnesium oxides, whereas slower cooling results in a higher CaO concentration and more variable SiO_2_ values.

### 3.4. XRD Results—Cement Synthesis

**Figure 4:**
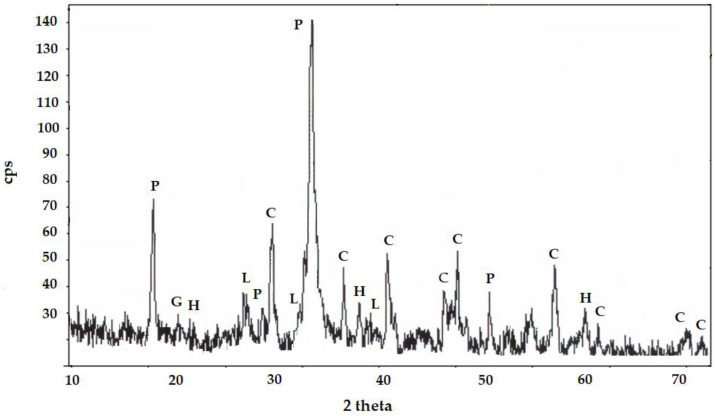
X-ray diffraction pattern of the sample with labeled crystalline phases. Crystalline phase labeling: P—Ca(OH)_2_; G—CaSO_4_·2H_2_O; L—βC_2_S; C—CaCO_3_; H—Mg_6_[(OH)_8_/Si_4_O_10_].

Before commencing experiments aimed at asbestos removal, detailed mineralogical studies were conducted on asbestos cement, or Eternit—a cement–asbestos material widely used as roofing, with an estimated total amount reaching several million tons. Phase analysis, performed using X-ray diffraction (XRD), revealed that in addition to chrysotile—the primary component of asbestos—Eternit also contains significant amounts of portlandite and smaller traces of calcite The XRD analysis is additionally illustrated in Figure 4.

These studies showed that chrysotile content in the analyzed Eternit samples reached up to 20%. This result was further confirmed by a quantitative analysis conducted using the Rietveld method, which enabled precise determination of the material’s phase composition [16] (Figure 4).

The obtained results provide key information on the mineralogical composition of asbestos cement, essential for developing effective methods for its safe disposal. These data will enable the reduction of hazards associated with the presence of asbestos. This research represents a significant step toward developing solutions for asbestos elimination from everyday environments.

#### Quantitative Composition Analysis

The quantitative phase composition analysis of samples subjected to different cooling methods indicates significant differences in clinker phase content and the amorphous phase. The application of various cooling methods considerably affects the proportions of individual phases, which is reflected in their physicochemical properties.

For the tricalcium silicate phase (C_3_S), responsible for rapid hydration and the initial strength of the material, a clear predominance of its content is observed in slowly cooled samples. C_3_S content in these samples ranges from 53.86% to 76.97%, while in rapidly cooled samples, it reaches lower values, ranging from 49.7% to 74.6%. Slow cooling promotes the stabilization and growth of this phase, which can be attributed to the longer crystallization process and more uniform structural formation under slower cooling rates.

**Figure 5:**
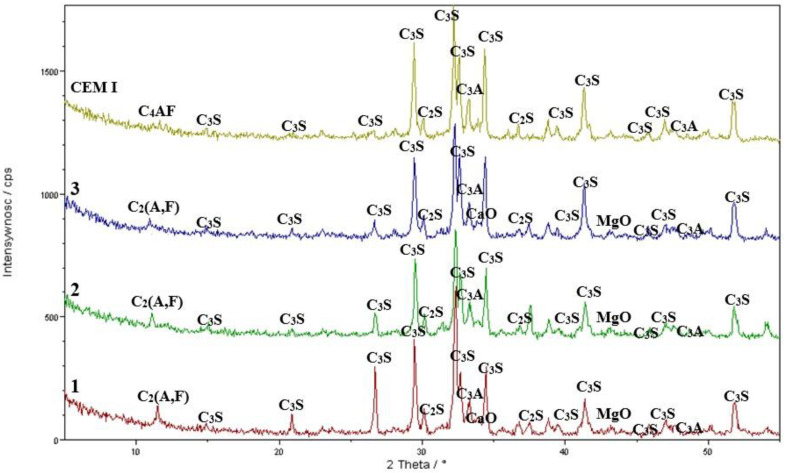
Composite X-ray diffraction pattern of the CEM I 42.5 R “Odra” cement sample, compared with the patterns of slowly cooled synthetic clinkers obtained from modified asbestos cement. Crystalline phase labeling: C_2_(A,F)—calcium aluminoferrate; C_2_S—dicalcium silicate; C_3_S—tricalcium silicate; C_3_A—tricalcium aluminate; CaO—calcium oxide; MgO—magnesium oxide.

An increase in the content of the dicalcium silicate phase (βC_2_S), characterized by slower hydration, is particularly noticeable in slowly cooled samples, where its content ranges from 4.39% to 22.31%. In rapidly cooled samples, the content of this phase is significantly lower, ranging from 3.61% to 18.3%. The slower cooling rate promotes the development of this phase, which may result from the extended phase transformation time, leading to greater stability of βC_2_S (Figure 5).

Similar trends can be observed for free lime (CaO). In slowly cooled samples, its content ranges from 1.3% to 1.6%, whereas in rapidly cooled samples, its concentration is lower, between 0.7% and 1.1%. The higher amount of free lime in slowly cooled samples may indicate better conditions for the crystallization of this component, resulting from prolonged exposure to high temperatures (Table 7).

The dicalcium alumino-ferrite phase -C_2_(A,F)- also shows distinct differences depending on the cooling method applied. In slowly cooled samples, the content of this phase ranges from 8.96% to 11.08%, whereas in rapidly cooled samples, it ranges from 6.2% to 8.2%. Slower cooling allows for better formation of this phase, likely due to a more uniform distribution of elements within the material structure.

For magnesium oxide (MgO), its content is significantly higher in slowly cooled samples, ranging from 2.58% to 4.19%, while in rapidly cooled samples, these values are lower, from 1.37% to 2.1%. Slow cooling promotes greater accumulation of MgO in the sample structure, possibly due to a more controlled rate of chemical reactions during cooling.

**Figure 6:**
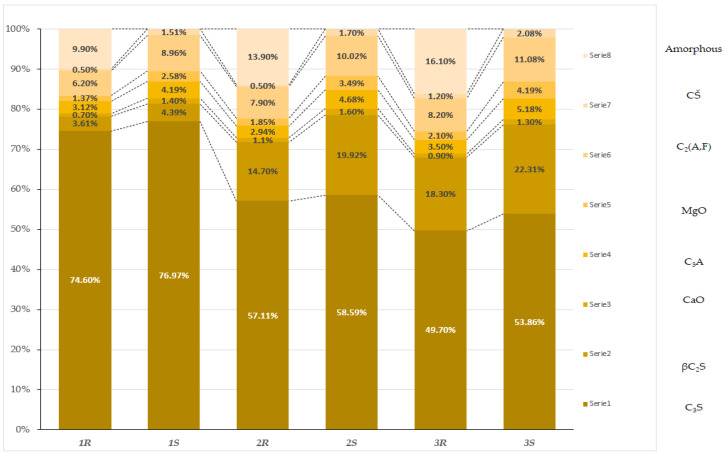
Graph of the percentage phase composition of cement samples after rapid and slow cooling. Description: 1R—sample 1 cooling rapidly (R); 1S—sample 1 cooling slowly (S); 2R—sample 2 cooling rapidly (R); 2S—sample 2 cooling slowly (S); 3R—sample 3 cooling rapidly (R); 3S—sample 3 cooling slowly (S).

Regarding the amorphous phase, it was noted that in rapidly cooled samples, its content ranged from 9.9% to 16.1%, whereas no amorphous phase was detected in slowly cooled samples. Rapid cooling favors the formation of this phase due to insufficient time for crystallization, resulting in the material being retained in an amorphous state. The differences in phase composition of samples due to the applied cooling methods are clear and significantly impact the microstructure and final properties of the material. Slow cooling promotes the development and stabilization of crystalline phases, while rapid cooling leads to a higher amorphous phase content and lower quantities of clinker phases (Figure 6).

### 3.5. Results of Physicochemical Properties of Cements

#### 3.5.1. Slowly Cooled Samples

An analysis of the presented results in Table 8 and Table 9 shows a systematic increase in flexural and compressive strengths at different maturation periods of cement paste samples made from slowly cooled clinkers. The study included measurements after 1, 2, 3, 7, 28, and 90 days from paste preparation. In each sample, strength increased with the maturation time of the cement paste, which is a typical phenomenon for Portland cement. The highest compressive strength, at 64.3 MPa, was obtained in the CEM I sample after 90 days, with flexural strength in the same sample reaching 8.3 MPa. A similar strength increase was also observed in the other samples, although their final values were slightly lower. For example, Sample 1 achieved a compressive strength of 54.5 MPa and a flexural strength of 9.0 MPa, while Sample 2 showed a compressive strength of 59.5 MPa and a flexural strength of 9.3 MPa after 90 days. Sample 3, which exhibited the lowest values compared to the others, reached a compressive strength of 50.4 MPa and a flexural strength of 7.5 MPa after the same maturation period. All obtained cements from modified molten asbestos cement had similar strengths to the commonly used CEM I 42.5 cement.

The setting time of the types of cement also differed between samples. The CEM I sample began setting after 2.45 h and completed setting after 5 h. For Sample 1, the initial setting time was 2 h, and the process concluded after 4 h. Sample 2 had the fastest initial setting time, at 1 h, with the process concluding after 2.55 h. Sample 3 began setting after 2 h, with setting completed after 3.45 h. These differences may result not only from the variable phase composition but also from differences in water-to-cement (W/C) ratios, which can influence the rate of chemical reactions occurring in the cement paste.

The flow of the cement paste mix, indicating its plasticity, varied between samples. The highest flow, at 190 mm, was observed in Sample 1, which had a W/C ratio of 0.50. In comparison, Sample CEM I, with a W/C ratio of 0.45, had a flow of 172 mm. Sample 2, with the same water-to-cement ratio as CEM I, exhibited a smaller flow of 165 mm. The smallest flow, at 160 mm, was observed in Sample 3, which also had a W/C ratio of 0.50.

Based on the data analysis, it can be concluded that all samples show a typical increase in strength as the cement paste matures. The highest strength was achieved by Sample CEM I, indicating the high quality of this mix. Samples 1 and 2, despite slightly lower compressive strength values, demonstrated good plasticity, which may enhance workability and homogenization. Sample 3, although showing the lowest flow values, achieved the lowest compressive and flexural strengths, likely due to its higher water-to-cement ratio.

Analyzing the results of linear change tests for cement paste samples, varied behaviors can be observed depending on the mix composition and curing conditions. Sample CEM I is characterized by predominant shrinkage, which gradually increases over time. On the first day, linear changes measure 0.00 mm/m, indicating no noticeable deformation. However, by the third day, the paste shows slight shrinkage at −0.02 mm/m, with the process continuing until reaching −0.07 mm/m after 90 days. This indicates a gradual reduction in sample volume, typical of this material type, which undergoes shrinkage due to water loss and cement hydration processes.

In contrast to CEM I, Sample 1 tends toward expansion. On the first day, the linear change value is 0.01 mm/m, indicating a slight increase in volume. In the following days, the volume change process intensifies, especially after six days, when linear changes reach 0.05 mm/m. Expansion remains steady in the subsequent days, reaching 0.20 mm/m on the 90th day. These results suggest that, unlike CEM I, Sample 1 shows a tendency to increase in volume, possibly due to different hydration reaction conditions or the composition of the cement paste, making this material non-shrinking.

Sample 2 exhibits the strongest swelling among all tested cement pastes. On the first day, linear changes measure 0.16 mm/m, increasing to 0.19 mm/m on the second day. After six days, the expansion intensifies, reaching 0.25 mm/m, with further increases in the following days. The maximum deformation value is observed on day 90, at 0.42 mm/m. This behavior in Sample 2 may indicate exceptionally intense swelling, possibly due to a higher water-to-cement ratio or other factors affecting paste volume growth, mainly related to the clinker phase composition. This material falls within the range of slightly expansive binders.

Sample 3 shows stable and minimal linear changes for most of the curing period. On the first day, changes are 0.01 mm/m, and in the following days, values remain close to zero, indicating minimal deformation in the early stages. Only after 28 days is there an increase in linear changes to 0.06 mm/m, with deformations reaching 0.31 mm/m by day 90. Sample 3, thus, tends to a delayed expansion, which intensifies only after the first month.

The obtained results indicate various behaviors of cement pastes. Sample CEM I predominantly exhibits shrinkage, while Samples 1, 2, and 3 are characterized by swelling, with the greatest expansion observed in Sample 2. Each sample responds differently depending on composition and hydration conditions, leading to distinct linear change values over time.

#### 3.5.2. Rapidly Cooled Samples

In the studies on the physicochemical properties of rapidly cooled cements, particular attention was paid to the analysis of flexural and compressive strength at different curing periods, as these properties are crucial for assessing the material’s durability and performance in construction. Samples were tested after 1, 3, 7, 28, and 90 days, allowing for an understanding of the dynamics of strength changes over time. The results of these studies are presented in Table 10.

For the cement designated as CEM I, 1 day after initiating the curing process, the flexural strength was 3.2 MPa, and the compressive strength was 9.5 MPa. By 7 days, the increase was significant, reaching 8.5 MPa (flexural) and 41.4 MPa (compressive). Maximum values were recorded after 90 days, with flexural strength at 9.2 MPa and compressive strength at 50.8 MPa. These results indicate good strength progression over time, typical for high-quality Portland cements.

The setting time of cement is also of particular relevance for practical applications. For CEM I, the initial setting time was 2.45 h, and the final setting occurred after 5 h, placing this material within the standard setting time range.

The other samples analyzed also showed a similar upward trend in mechanical strength, although differences in initial results indicate some variations in cement microstructure. For example, Sample 2 had lower initial results compared to CEM I—with a flexural strength of 2.4 MPa and compressive strength of 6.9 MPa after 1 day. Nevertheless, after 90 days, flexural strength reached 9.2 MPa, and compressive strength was 49.4 MPa, indicating good mechanical properties after 90 days. It is noteworthy that Sample 2 had a slightly shorter setting time—the process began after 2.30 h and was completed after 4.50 h, making it a faster-setting material compared to CEM I.

Sample 3 exhibited the highest initial flexural and compressive strengths—3.1 MPa and 11.7 MPa, respectively, after 1 day. After 90 days, flexural strength reached 9.5 MPa, and compressive strength reached 52.5 MPa, making this sample the strongest in terms of compressive strength among those tested. In this case, a faster setting process was also observed, beginning after 2.20 h and concluding after 4.15 h, which may suggest the suitability of this material for conditions requiring rapid structural adaptation of the cement.

The analysis of cement flow results also provides interesting insights. The flow value for CEM I was 172 mm, which falls within standard limits, while Samples 2 and 3 exhibited values of 162 mm and 183 mm, respectively. These values indicate stable rheological properties, meaning that these cements possess suitable plasticity and can be easily worked, which is crucial for concrete applications in construction [22]. The water-to-cement ratio (W/C) maintained at 0.45 for all samples indicates an optimal balance between water and cement contents, necessary to achieve appropriate mechanical properties and a proper hydration process [22].

The results for rapidly cooled cements demonstrate their high efficiency and significant strength increase over time. Special attention should be paid to the faster setting process of some samples and their varied strength shortly after the initiation of the curing process. The stability of rheological properties and the optimal W/C ratio further confirm their high quality and potential application in demanding construction conditions (Table 10).

The analysis of linear changes in rapidly cooled cement samples provides essential information about their dimensional stability during the curing process, which is particularly significant for applications where volumetric changes can lead to internal stresses and impact the structure’s durability [23]. Linear change measurements were conducted over 90 days, allowing for observation of the tendency for the material to shrink or expand in different maturation phases. The results of these studies are presented in Table 11.

For CEM I cement, linear changes were relatively small and showed a tendency toward material shrinkage in the initial curing phases. After 1 day, the linear change was 0.00 mm/m. On day 3, a slight shrinkage of −0.02 mm/m was observed, which continued on day 6 (−0.01 mm/m) and day 7 (−0.04 mm/m). By day 9, the change was 0.00 mm/m, suggesting temporary dimensional stability. From day 14 to day 90, a slight shrinkage of -0.05 mm/m was noted, indicating final stabilization of the volumetric changes in CEM I cement. These results suggest that CEM I is relatively dimensionally stable after an initial period of shrinkage in the first few days following the hydration process.

The other samples exhibited varied linear change tendencies. Sample 1 showed a linear change of 0.08 mm/m after 1 day, indicating slight expansion. However, over time, the material stabilized, and linear changes in subsequent days became progressively smaller—0.07 mm/m after 3 days and 0.03 mm/m after 6 days. By day 7, further reductions in linear changes were observed, reaching 0.02 mm/m, and from day 9 to day 90, the changes were only 0.01 mm/m, suggesting that the material achieved dimensional stability.

Sample 2 exhibited a similar initial behavior, but the linear changes were somewhat larger. After 1 day, a linear change of 0.07 mm/m was observed, which increased in the following days. After 6 days, it reached 0.10 mm/m, and after 7 days, it rose to 0.14 mm/m, indicating greater expansion of the material compared to Sample 1. After 9 days, the linear change value dropped to 0.10 mm/m, and from day 14, it remained steady at 0.07 mm/m until day 90. These results suggest that Sample 2, despite its initial greater expansion, demonstrated stabilization in the later period.

Sample 3, similar to CEM I, showed a tendency to shrink. After 1 day, the linear change was 0.00 mm/m, while a shrinkage of −0.01 mm/m was recorded on days 3 and 6. After 7 days, a slight expansion of 0.02 mm/m was noted, but by day 9, the material shrank again (−0.01 mm/m), and from day 14 to day 90, linear changes remained at −0.05 mm/m. These results indicate a similar tendency to shrink as seen in CEM I, suggesting similar physicochemical and mechanical properties in these samples.

The analysis of linear changes shows diverse behavior among the tested samples, with CEM I and Sample 3 tending to shrink, while Sample 1 demonstrated stabilization after initial expansion, and Sample 2, despite greater expansion, ultimately achieved dimensional stability. These results suggest that these materials hold potential for applications where controlling volumetric changes is critical for structural durability (Table 11).

The results, including the increase in compressive strength and linear changes over time, are visualized in the relevant figures. The models were developed in accordance with general statistical principles (see, e.g., [24,25]). Temporal dynamics were computed using the least mean squares errors (LMSE) method, assuming nonlinearity and logarithmic trends. The models’ equations, along with the values of the coefficient of determination (R^2^), are presented in the figures.

**Figure 7:**
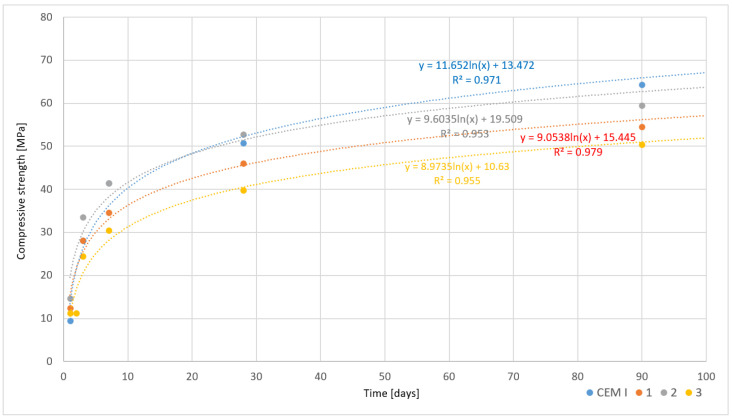
Changes in compressive strength as a function of time for different cement pastes—slowly cooled samples.

**Figure 8:**
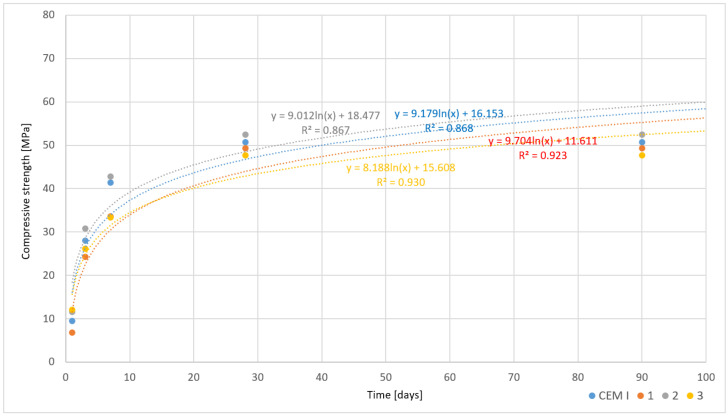
Changes in compressive strength as a function of time for different cement pastes—rapidly cooled samples.

The graphs presented below illustrate the results of compressive strength tests for cements obtained from chemically modified asbestos cement, which were cooled under two different conditions—slowly (Figure 7) and rapidly (Figure 8). Each graph also includes data for the reference cement CEM I, allowing for a comparison of the results obtained for the innovative cement materials with traditional Portland cement.

Analyzing the reference cement (CEM I), it can be observed that regardless of the cooling method, it exhibits the highest compressive strength, achieving values exceeding 60 MPa by the end of the 90-day curing period in both cases. This is characteristic of Portland cement, which demonstrates stable and predictable mechanical properties due to the prolonged hydration process, reaching a state of hydration equilibrium within about 28 days, after which further strength gain is minimal. The lack of significant differences in results between slowly and rapidly cooled samples suggests that the cooling process has a negligible impact on the strength development of this type of cement, likely due to its more homogeneous chemical structure and predictable hydration properties.

In the case of cements obtained through the melting of chemically modified asbestos cement (Samples 1, 2, 3), the results are more varied and clearly depend on the cooling method. The graph depicting slowly cooled samples shows a gradual increase in compressive strength, with relatively small differences between the individual samples. Notably, Sample 1 stands out with the highest strength values, comparable to the reference cement, suggesting that the chemical modifier in this sample positively influences the development of the hydration structure during slow cooling. These samples achieve higher strength values compared to those cooled rapidly, which can be attributed to a more uniform and controlled hydration process that promotes the formation of stable phases, such as calcium silicate, responsible for increased mechanical strength.

In contrast, the rapidly cooled samples exhibit greater variability in results. Sample 3, in particular, stands out with a lower compressive strength, which may be a consequence of disrupted hydration processes that, under rapid cooling conditions, are more prone to internal thermal stresses. High cooling rates can lead to the formation of microcracks or unstable hydration products, negatively impacting the mechanical properties of the cement. Although Samples 1 and 2 achieve higher strength values than Sample 3, they also present lower results compared to their slowly cooled counterparts. This indicates that rapid cooling limits the full utilization of the hydration potential of the modified cements, resulting in a less homogeneous structure and reduced strength.

An interesting relationship is also observed in the difference in the development of early strength. The graph depicting rapidly cooled samples shows a faster increase in strength during the initial days of setting compared to slowly cooled samples. This may result from the intensification of hydration processes in the early phases of the process; however, the rapid growth of early strength does not translate into higher final values after 90 days. This suggests that rapid cooling may favor the formation of unstable hydration products, which contribute to a temporary increase in strength but do not provide long-term structural stability.

The reference cement CEM I exhibits stable results regardless of the cooling method, attributed to its predictable microstructural composition and well-known hydration processes. In the case of chemically modified cements, the cooling method significantly influences the final mechanical properties. Slow cooling promotes the uniform development of the hydration structure, leading to higher and more consistent strength results, whereas rapid cooling, although it may lead to faster early strength gain, negatively impacts long-term strength. This is particularly evident in samples such as Sample 3, which shows significantly lower results compared to the others.

**Figure 9:**
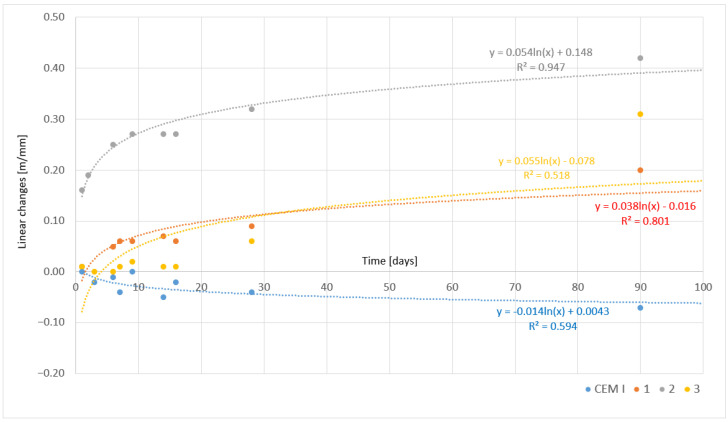
Linear changes in cement pastes as a function of time—slowly cooled samples.

**Figure 10:**
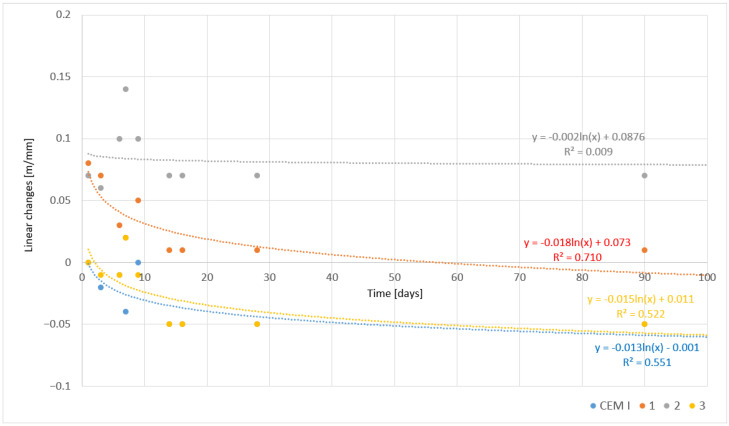
Linear changes in cement pastes as a function of time—rapidly cooled samples.

The following graphs illustrate the linear changes in cements as a function of time, depicting the deformations occurring in the materials due to hydration processes and chemical reactions. The study included both the reference cement CEM I and chemically modified cements obtained through the melting of modified asbestos cement. The cement samples were cooled under two different conditions—slowly (Figure 9) and rapidly (Figure 10). Analyzing these results provides valuable information on the volumetric stability, expansion tendencies, and shrinkage of the materials tested, which is crucial for assessing their potential applications in construction.

The reference cement CEM I, in both slowly and rapidly cooled samples, exhibits minimal linear deformations that remain within the range of negative values, indicating a slight tendency to shrink. Throughout the 90-day study period, these values do not exceed −0.05 mm/m. The shrinkage of this type of cement is mainly due to water loss during hydration and evaporation. The small linear deformation values indicate a stable microstructure in CEM I, where shrinkage processes quickly balance out through hydration products. The volumetric stability of the reference cement, regardless of the cooling method, reflects its predictable mechanical properties, making it suitable for engineering applications [26].

Chemically modified cements obtained through the melting of modified asbestos cement exhibit significantly greater linear changes compared to the reference cement. These deformations result from intense hydration processes and chemical reactions associated with the presence of modifiers, which affect the expansion of the material. The results of the study clearly indicate that the linear changes of these cements vary depending on the cooling method applied, as can be observed in both graphs.

In the case of slowly cooled samples, the largest linear deformations occur in Sample 1, reaching maximum values around 0.4 mm/m. Such significant expansion of the material results from the intense development of hydration products, such as ettringite and other compounds, which lead to the expansion of the cement structure. Slow cooling promotes a more uniform and complete hydration process, allowing for the gradual but stable development of hydration phases responsible for volume increase. After approximately 30 days, the rate of deformation growth begins to decrease, indicating stabilization of the material’s microstructure. Similar phenomena can be observed in Samples 2 and 3, although their deformations are smaller, oscillating around 0.2–0.3 mm/m. This suggests that other modifier components in these samples contribute to less intense expansion processes, which may result from differing rates of hydration or the formation of other hydration products.

Conversely, for the rapidly cooled samples, we observe smaller linear deformations, indicating limited development of material expansion. Sample 1 shows maximum values of 0.15 mm/m, which suggests restricted growth of hydration phases, including ettringite, due to the quicker cessation of hydration processes. Rapid cooling limits the expansion of the cement, which may promote volumetric stability; however, it simultaneously hinders the full development of mechanical properties that could arise from a longer and more controlled cooling process. Samples 2 and 3, in the rapidly cooled group, exhibit even lower deformation values, not exceeding 0.1 mm/m. The limitation of deformations can be attributed to the faster closure of hydration processes, which may lead to a more homogeneous and stable cement structure, but at the same time, it may restrict its potential mechanical properties in the longer term.

An interesting relationship observed in both cases is the difference in the rate of achieving maximum deformation values. In the slowly cooled samples, this process is more dynamic, suggesting that slow cooling promotes a more intense development of hydration phases responsible for material expansion. In contrast, the rapidly cooled samples exhibit linear changes that are more stretched over time, indicating less intense but more stable hydration processes [27].

The reference cement CEM I shows stable and minimal linear changes, indicating its good volumetric stability and predictable mechanical properties, regardless of the cooling method applied. Chemically modified cements obtained through the melting of modified asbestos cement exhibit significantly greater linear changes, especially in the early hydration phases, with their intensity depending on the cooling method. Slow cooling promotes larger deformations due to the more complete development of hydration phases responsible for the material’s expansion. Conversely, rapid cooling limits these changes, which may provide greater structural stability but simultaneously restricts the full development of the properties of the modified cements. These conclusions are significant for construction practice, as the choice of cooling method and the composition of modified cement can determine its long-term durability and volumetric stability under various operating conditions.

## 4. Conclusions

1. Cements modified with asbestos waste achieved a compressive strength comparable to CEM I cements. The compressive strength after 90 days was close to the values for CEM I, being lower by 2–15% depending on the cooling method applied. Additionally, cements containing asbestos demonstrated potential for use in construction due to maintaining standard physico-chemical parameters.
2. The cooling method of the clinkers significantly affected the hydraulic and mineralogical properties of the cements. Slow cooling promoted a higher content of tricalcium silicate (C_3_S), reaching up to 76.97%, while rapid cooling favored a higher content of amorphous phases (ranging from 9.9% to 16.1%). Amorphous phases can intensify the hydration process, leading to distinct mechanical and volumetric properties of the cement.
3. Asbestos-modified cements exhibited varying linear changes, from shrinkage of −0.07 mm/m (CEM I) to expansion of 0.42 mm/m (sample 2). This phenomenon resulted from the use of different clinker cooling methods, which influenced the phase composition and hydration processes.
4. The implementation of asbestos waste, particularly chrysotile, in Portland cement production contributes to reducing the demand for natural raw materials and lowering CO_2_ emissions, making this process environmentally and economically beneficial. Research confirms that asbestos waste can be effectively utilized as a mineral additive, enabling the production of cements with mechanical properties comparable to conventional cements. Furthermore, the elimination of asbestos fibers from circulation significantly improves health safety while supporting sustainable development and effective waste management.
5. It is recommended to conduct detailed studies on the hydraulic and chemical activity of cements modified with asbestos waste, as well as their long-term environmental and health impacts. Analyzing calorimetric and hydration properties will allow for a deeper understanding of the potential of these materials in various industrial applications.

## Figures and Tables

**Table 1:** Chemical composition of the calcium carbonate sample.

Component	Content [% by Mass]
CaCO_3_	99
HCI	0.005
N	0.04
Cl	0.001
SO_4_	0.05
Mg	0.05
K	0.01
Na	0.05
Fe	0.001

**Table 2:** Chemical composition of the silica gel sample.

Component	Content [% by Mass]
SiO_2_	99.7
Na_2_O	0.1
Al_2_O_3_	0.05
Fe_2_O_3_	0.03
TiO_2_	<0.01
Organic impurities	<0.01
Moisture (H_2_O)	0.1

**Table 3:** Chemical composition of the asbestos cement sample.

Component	Content [% by Mass]
SiO_2_	25.82
Al_2_O_3_	4.11
Fe_2_O_3_	3.52
TiO_2_	0.31
CaO	56.87
MgO	6.93
K_2_O	0.59
Na_2_O	0.15
SO_3_	1.70

**Table 4:** Chemical composition of selected Portland cement.

Component	Type of Portland Cement		
	“CHEŁM” CEM I 42.5 N-HSR/NA	“GÓRAŻDŻE” CEM I 42.5R	“ODRA” CEM I 42.5R
	Content [% by Mass]		
SiO_2_	21.10	21.48	18.50
Al_2_O_3_	3.23	5.10	5.50
Fe_2_O_3_	4.51	2.75	2.70
CaO	64.65	64.90	64.00
MgO	1.13	1.44	1.60
SO_3_	3.08	2.53	2.10
K_2_O	0.43	0.83	1.00
Na_2_O	0.20	0.14	0.10
Cl^−^	0.047	0.08	0.028
Na_2_Oeq	0.48	-	-

**Table 5:** Composition of raw mix formulations for Portland cements.

Modified Raw Material Samples (Formulations for Cement Clinker)			
Symbol	1	2	3
Composition	Eternit—65% CaO—30% SiO_2_—5%	Eternit—75% CaO—22% SiO_2_—3%	Eternit—85% CaO—15%

**Table 6:** Chemical composition of rapidly and slowly cooled clinkers.

Clinkers						
Type of Cooling	1		2		3	
	Rapid	Slow	Rapid	Slow	Rapid	Slow
Chemical Composition [Mass %]						
SiO_2_	21.29	20.83	21.02	18.84	19.99	21.44
Al_2_O_3_	5.57	5.39	6.27	4.09	4.18	2.22
Fe_2_O_3_	1.68	1.66	3.14	2.42	2.20	1.43
TiO_2_	0.22	0.23	0.24	0.18	0.18	0.12
CaO	67.19	68.62	62.20	68.77	63.74	69.38
MgO	3.75	2.68	7.03	5.65	9.67	5.32
K_2_O	<0.01	0.02	0.03	<0.02	<0.01	<0.01
Na_2_O	0.20	0.49	<0.01	<0.02	<0.01	<0.01

**Table 7:** Comparison of phase composition of cement samples depending on the cooling method applied.

Clinkers						
Type of Cooling	1		2		3	
	Rapid	Slow	Rapid	Slow	Rapid	Slow
Phase Composition [Mass %]						
C_3_S	74.60	76.97	57.11	58.59	49.7	53.86
βC_2_S	3.61	4.39	14.7	19.92	18.3	22.31
CaO	0.7	1.4	1.1	1.6	0.9	1.3
C_3_A	3.12	4.19	2.94	4.68	3.5	5.18
MgO	1.37	2.58	1.85	3.49	2.1	4.19
C_2_(A,F)	6.2	8.96	7.9	10.02	8.2	11.08
CŜ	0.5	1.51	0.5	1.7	1.2	2.08
Amorphous	9.9	-	13.9	-	16.1	-

**Table 8:** Results of physicochemical properties of slowly cooled cement.

Sample	Flexural/Compressive Strength in MPa [Days]						Setting Time [hours]		Flow [mm]	W/C
	1	2	3	7	28	90	Start	End		
CEM I	3.2/9.5	-	6.4/28.1	8.5/41.4	9.2/50.8	9.3/64.3	2.45	5.0	172	0.45
1	3.7/12.4	-	5.9/28.1	7.6/34.6	9.0/46.1	9.0/54.5	2.0	4.0	190	0.50
2	4.0/14.7	-	6.5/33.5	7.8/41.5	9.2/52.8	9.3/59.5	1.0	2.55	165	0.45
3	3.0/11.2	3.0/11.2	5.3/24.5	6.5/30.5	7.5/39.8	7.5/50.4	2.0	3.45	160	0.50

**Table 9:** Results of linear change tests for slowly cooled cements.

Sample	Linear Changes [mm/m]									
	Days									
	1	2	3	6	7	9	14	16	28	90
CEM I	0.00	-	−0.02	−0.01	−0.04	0.00	−0.05	−0.02	−0.04	−0.07
1	0.01	-	-	0.05	0.06	0.06	0.07	0.06	0.09	0.20
2	0.16	0.19	-	0.25	-	0.27	0.27	0.27	0.32	0.42
3	0.01	-	0.00	0.00	0.01	0.02	0.01	0.01	0.06	0.31

**Table 10:** Results of physicochemical properties of rapidly cooled cements.

Sample	Flexural/Compressive Strength in MPa [Days]						Setting Time [hours]		Flow [mm]	W/C
	1	2	3	7	28	90	Start	End		
CEM I	3.2/9.5	-	6.4/28.1	8.5/41.4	9.2/50.8	9.2/50.8	2.45	5.00	172	0.45
1	2.4/6.9	-	5.3/24.3	7.4/33.6	9.2/49.4	9.2/49.4	2.30	4.50	162	0.45
2	3.1/11.7	-	6.0/30.0	8.2/42.1	9.5/52.5	9.5/52.5	2.20	4.15	183	0.45
3	3.6/12.1	-	5.6/26.2	6.9/33.4	8.8/47.7	8.8/47.7	3.20	4.50	161	0.45

**Table 11:** Results of linear change tests for rapidly cooled cements.

Sample	Linear Changes [mm/m]									
	Days									
	1	2	3	6	7	9	14	16	28	90
CEM I	0.00	-	−0.02	−0.01	−0.04	0.00	−0.05	−0.05	−0.05	−0.05
1	0.08	-	0.07	0.03	0.02	0.05	0.01	0.01	0.01	0.01
2	0.07	-	0.06	0.10	0.14	0.10	0.07	0.07	0.07	0.07
3	0.00	-	−0.01	−0.01	0.02	−0.01	−0.05	−0.05	−0.05	−0.05

## Data Availability

The original contributions presented in this study are included in the article. Further inquiries can be directed to the corresponding author.
